# Effect of curcumin supplementation on exercise-induced muscle damage: a narrative review

**DOI:** 10.1007/s00394-022-02943-7

**Published:** 2022-07-13

**Authors:** K. Nanavati, K. Rutherfurd-Markwick, S. J. Lee, N. C. Bishop, A. Ali

**Affiliations:** 1 School of Sport, Exercise, and Nutrition, Massey University, Auckland, New Zealand; 2 School of Health Sciences, Massey University, Auckland, New Zealand; 3 School of Food and Advanced Technology, Massey University, Auckland, New Zealand; 4 School of Sport, Exercise and Health Sciences, Loughborough University, Loughborough, UK

**Keywords:** Curcumin, Pharmacokinetics, Inflammation, Muscle soreness, Oxidative stress, Antioxidant

## Abstract

Curcumin, a natural polyphenol extracted from turmeric, is a potent antioxidant and anti-inflammatory agent. In the past few decades, curcumin’s ability to impact chronic inflammatory conditions such as metabolic syndrome, arthritis, and cancer has been widely researched, along with growing interest in understanding its role in exercise-induced muscle damage (EIMD). EIMD impacts individuals differently depending on the type (resistance exercise, high-intensity interval training, and running), intensity, and duration of the exercise. Exercise disrupts the muscles’ ultrastructure, raises inflammatory cytokine levels, and can cause swelling in the affected limb, a reduction in range of motion (ROM), and a reduction in muscular force-producing capacity. This review focuses on the metabolism, pharmacokinetics of various brands of curcumin supplements, and the effect of curcumin supplementation on EIMD regarding muscle soreness, activity of creatine kinase (CK), and production of inflammatory markers. Curcumin supplementation in the dose range of 90–5000 mg/day can decrease the subjective perception of muscle pain intensity, increase antioxidant capacity, and reduce CK activity, which reduces muscle damage when consumed close to exercise. Consumption of curcumin also improves muscle performance and has an anti-inflammatory effect, downregulating the production of pro-inflammatory cytokines, including TNF-*α*, IL-6, and IL-8. Curcumin may also improve oxidative capacity without hampering training adaptations in untrained and recreationally active individuals. The optimal curcumin dose to ameliorate EIMD is challenging to assess as its effect depends on the curcumin concentration in the supplement and its bioavailability.

## Introduction

Curcumin, chemically known as 1,7-bis(4-hydroxy-3-methoxyphenyl)-1,6-heptadiene-3,5-dione or diferuloylmethane [1], is isolated from the plant *Curcuma longa* [2]*.* Over the past few decades, curcumin has been widely researched for its antioxidant and anti-inflammatory properties in numerous chronic and malignant diseases such as metabolic syndrome [3], arthritis [4, 5], and cancer [6–8]. However, there has been a growing interest in understanding the effect of curcumin on exercise-induced muscle damage (EIMD) in recent years. EIMD affects all individuals depending upon the type, intensity, and duration of the exercise they undertake and training status of the individual [9, 10]. Resistance training [11], high-intensity interval training [12], trail running [13, 14], and downhill running [15] contribute to EIMD, leading to ultrastructural muscular disruption and an increase in inflammatory cytokine levels. Swelling of the affected limb, decreased range of motion (ROM), and impaired muscle force-producing capacity which can result from EIMD are undesirable [16–18].

Curcumin has been shown to attenuate muscle soreness, improve performance, reduce blood levels of inflammatory markers, and enhance endogenous oxidative capacity post-exercise [19–26]. Muscle damage is prominent post-exercise due to the release of prostaglandins under the influence of cyclooxygenase (COX-1 and COX-2), which contributes to redness, swelling, and pain at the site of damage [27, 28]. Curcumin downregulates the expression of COX-2 and thus decreases the release of prostaglandins [29] which in turn reduces muscle damage [19–23]. Exercise, and production of prostaglandins under the influence of COX-2, leads to increased membrane permeability [30, 31] and releases creatine kinase (CK) into the interstitial fluid and then circulation via the lymphatic system [32] and indicates muscle damage. Although research on the chronic effects of curcumin supplementation is limited, data so far have not demonstrated any ergolytic effects as observed following supplementation with other natural antioxidants such as vitamin C and E supplementation. A detailed review [36] on the effects of vitamin C and vitamin E supplementation on exercise performance suggests that these antioxidant supplementations may impair neuromuscular adaptation by affecting muscle mitochondrial biogenesis and muscle hypertrophy [33].

Clinical trials indicate consumption of curcumin close to exercise downregulates cyclooxygenase that influences membrane permeability [31] and offers a membrane-protective effect by altering the structure of the cell membrane to improve its integrity [34]. Curcumin also helps in reducing inflammation by hindering the activation of nuclear factor-kappa B (NF-κB), suppressing the activation and phosphorylation of Janus kinase/signal transducers and activators of transcription (JAK/STAT) proteins, and inhibiting mitogen-activated protein kinase (MAPK) signalling that releases inflammatory markers such as tumour necrosis factor-alpha (TNF-*α*), interleukin-8 (IL-8), and interleukin-6 (IL-6) at the site of damage [35]. In addition, the downregulation of NF-κB can also lead to the elevation of antioxidant responses by activation of nuclear factor erythroid 2-related factor 2 (NRF2) [36]. NRF2 regulates the synthesis of antioxidant proteins that protect against oxidative damage triggered by injury and inflammation [37].

Curcumin can be tolerated without any associated toxicity at 8000 mg/day [38]. However, poor aqueous solubility [39] and low bioavailability [40] of curcumin have led to the development of different curcumin formulations such as nanoparticles [41], phytosomes [42], micelles [43], and phospholipid complexes [44]. Each formulation contains varying levels of curcuminoids and has a different rate of absorption, making it difficult to conclude a single recommended dose. Nevertheless, different curcumin formulations have been shown to effectively reduce EIMD and inflammation in doses varying from 90 mg/day to 5000 mg/day [21, 25]. This narrative review evaluates the different curcumin formulations and their effect on EIMD, inflammation, and oxidative markers.

## Methods

The databases SCOPUS, Medline (PubMed), and Web of Science (WOS) were searched using a mix of Medical Subject Headings (MeSH) and free words for key concepts related to curcumin, muscle, exercise, inflammation, recovery, along with bioavailability of curcumin as follows: (“curcumin” OR “turmeric”) AND (“muscle damage” OR “delay onset muscle soreness” OR “DOMS” OR “inflammation” OR “inflammatory” OR “inflammatory markers” OR “oxidative stress”) AND (“exercise”). For articles on other natural antioxidants and their effect on exercise performance, search terms included (“antioxidants”), (“vitamin E and C”), (“tart cherry juice”), (“natural extracts”) AND (“exercise” OR “exercise-induced muscle damage” OR “exercise performance’’), between December 2020 and March 2021. Only full-text articles (written in English) describing human trials were included for review.

### Curcumin metabolism

**Fig. 1:**
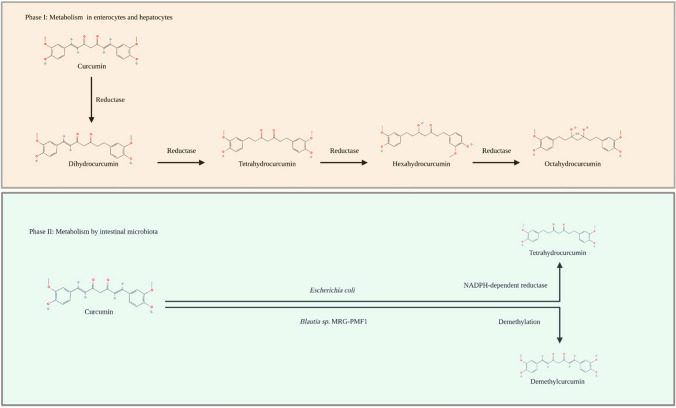
Metabolic pathways of curcumin

The bioavailability of curcumin is low due to its water insolubility and poor metabolism in the small intestine and liver, where it undergoes extensive reductive and conjugative metabolism and is finally eliminated through the gall bladder [45]. The metabolism of curcumin can be divided into two phases (Fig. 1). Phase I comprises the reduction of its double bonds to dihydrocurcumin, tetrahydrocurcumin, hexahydrocurcumin, and octahydrocurcumin by reductases in enterocytes and hepatocytes [40]. For phase II, both curcumin and its metabolites from phase I undergo conjugation with sulphate at its phenolic site in the hepatic and intestinal cytosol. In addition, metabolites also undergo glucuronidation via UDP-glucuronosyltransferase in the intestinal and hepatic microsomes [40]. Alternatively, curcumin can be metabolised by intestinal microbiota, such as *Escherichia coli* to tetrahydrocurcumin with the help of an NADPH-dependent reductase and by *Blautia *sp. MRG-PMF1 (anaerobic bacterial strain) that facilitates curcumin demethylation to form demethylcurcumin and bis-demethylcurcumin [46].

### Pharmacokinetics of curcumin supplements

Absorption, distribution, hepatic and intestinal metabolism, and excretion regulate the bioavailability of ingested curcumin [45]. In addition, the physicochemical properties of each curcumin formulation and the dose, along with its rate of degradation in the lumen, lipophilicity, and gastric emptying time, help determine the body’s pharmacokinetics [47].

Some studies have assessed plasma curcumin levels after hydrolysis of blood plasma samples, however, such hydrolysis prior to extraction masks the amount of free, bioactive curcumin and total curcuminoids as compared to non-hydrolysed samples [50]. When treated with the enzymes *β*-glucuronidase and sulfatase, curcumin generates glucuronide curcumin and curcumin sulphate, which are the primary circulating forms of curcumin but are physiologically inactive conjugates. This obscures the free bioactive curcumin and overestimates the amount of curcumin detected, thus providing incorrect and misleading results regarding the bioavailability of the formulation [48]. This highlights the importance of reporting free curcumin in the plasma without hydrolysis of the sample. Plasma samples obtained from studies involving Theracurmin [41], Meriva [42], and NovaSol [43] were all hydrolysed using *β*-glucuronidase/sulfatase before analysis and thus the bioavailability results should be viewed with caution.

#### Overview of bioavailability of different curcumin formulations

Information on dosage, study type, and study population of six different formulations, namely Theracurmin [41], Meriva [42], NovaSol [43], CurQfen [49], Longvida [44], and Curcumin C3 Complex [50], along with their pharmacokinetic parameters are presented in Tables 1 and 2 [47]. The evidence from human trials suggests that formulating curcumin improves systemic exposure and increases area under curve (AUC) and maximum blood concentration of curcumin, and thus, increases the bioavailability of curcumin (*C*_max_) [41–44, 49, 50].

**Table 1:** Composition of different curcumin formulations

Formulation name	Formulation		
	Technology	Curcuminoid concentration	Ingredients
Theracurmin [41]	Colloidal nanoparticles	12%	12% curcuminoids, 46% glycerine, 4% gum ghatti, 38% water
Meriva® [42]	Phytosome	18–20%	Curcumin, soy lecithin, microcrystalline cellulose
NovaSol [43]	Liquid micelles	6%	93% Tween 80, and 7% curcumin powder
CurQfen [49]	Soluble fibre blend	Not defined	Fenugreek soluble fibre blend, and 40% curcumin
Longvida® [44]	Solid lipid curcumin particle	20–30%	Solid lipid curcumin particle lipids, phosphatidylcholine, and 20% curcumin
Curcumin C3 Complex® + Bioperine [50]	Not applicable	Not defined	Bioperine and curcuminoids

**Table 2:** Pharmacokinetic parameters of curcumin from the different curcumin-based formulations and reference (unformulated curcumin) in human studies

Formulation	Clinical study design	Population	Intervention	Dose	Sample hydrolysis	*C*_max_ (ng/mL)	*T*_max_ (h)	AUC_0–t_ (ng h/mL)
Theracurmin [41]	Randomised, crossover	Asian 14 (8 males, 6 females) 44.1 ± 8.5 years	Formulation^a^	30 mg theracurmin	Hydrolysed	29.5 ± 12.9	1	113 ± 61^*^
			Control^a^	30 mg curcumin powder		1.8 ± 2.0	6	4.1 ± 7.0^*^
Meriva® [42]	Randomised, double-blind, crossover	Caucasian 9 (8 males, 1 female)	Formulation^b^	376 mg curcumin	Hydrolysed	206.9 ± 54.9	2.7 ± 0.3	1336.0 ± 357.1^*^
			Control^b^	1799 mg curcumin		14.4 ± 4.2	6.9 ± 2.2	202.8 ± 53.8^*^
NovaSol [43]	Randomised, single‐blind crossover	Caucasian 23 (10 males, 13 females) 23 ± 3 years	Formulation^a^	500 mg curcuminoids	Hydrolysed	1189.1 ± 518.7	1.1 ± 0.4	4474.7 ± 1675.2^⁑^
			Control^a^	500 mg curcuminoids		2.6 ± 4.9	7.5 ± 8.2	24.1 ± 42.6^⁑^
CurQfen [49]	Crossover	Indian 8 (males) 25–50 years	Formulation^a^	600 mg curcumin	Not hydrolysed	0.4 ± 0.2 (µg/g)	1	8100 ± 287^*^ (µg h/g)
			Control^a^	1000 mg curcumin		0.02 ± 0.01 (µg/g)	0.5	510 ± 123^*^ (µg h/g)
Longvida® [44]	Randomised, double-blind, crossover	Indian 6 (males) 18–40 years	Formulation^b^	650 mg curcuminoids	Not hydrolysed	22.4 ± 1.9	2.4 ± 0.4	95.3 ± 4.6^*^
			Control^b^	650 mg curcuminoids		< 1	ND	ND
Curcumin C3 Complex® + Bioperine [50]	Randomised, crossover	Indian 8 (males) 20–26 years	Formulation^b^	2000 mg curcumin with bioperine	Not hydrolysed	180 ± 30	0.69 ± 0.07	80 ± 10^†^
			Control^b^	2000 mg curcumin		6 ± 5	1	4^†^

^a^ Mean ± standard deviation

^b^ Mean ± standard error of mean

^*^AUC_0–24_

^⁑^AUC_0–12_

^†^ AUC_0–6_

Control: unformulated curcumin, *AUC* area under the drug concentration–time curve, *C*_*max*_ maximum drug concentration, *T*_*max*_ time at maximum drug concentration, *ND* not defined

The use of gum ghatti in a water-soluble formulation called Theracurmin [41] led to a preparation of a stable water-soluble complex that contributed to colloidal dispersion and enhanced gastrointestinal absorption [41]. A dose of 30 mg of Theracurmin containing 3.6 mg curcuminoids showed a 27-fold improvement in its AUC_(0–6 h),_ with a *T*_max_ of 1 h. Another water-soluble curcumin: NovaSol [43] demonstrated the highest *C*_max_ along with 185-fold better bioavailability compared to its native form, with a single dose of 500-mg of curcuminoids. The improved relative bioavailability was contributed by the micellar-based curcumin formulation with Tween 80 (non-ionic surfactant and emulsifier) that could deliver most of the curcumin to the intestinal wall for absorption by escaping the phase separation in the gastrointestinal tract [43]. Schiborr et al. [43] also observed that less than 0.2% of the oral dose of Novasol curcumin was excreted in urine within 24 h, and concluded that the remaining >98.8% of the ingested curcumin was either excreted via the bile and faeces or may have been distributed to body tissues where it may potentially exert biological activities. Meriva [42], a formulation using natural curcuminoids and lecithin (phosphatidylcholine phytosome complex of soy) in the ratio of 2:1 along with two parts of microcrystalline cellulose yielded the highest *T*_max_ at 2.7 ± 1 h for a dose of 376 mg of curcuminoids, and 29-fold higher curcuminoid absorption compared to the unformulated curcumin. Interestingly, the authors [42] concluded that lecithin favoured the bioavailability of demethoxycurcumin as its plasma content was found to be higher than curcumin itself, despite its low concentration in the formulation.

The formulation of fenugreek fibre and 40% curcumin called CurQfen [51] yielded the highest AUC over 24 h at a dose of 1500 mg (equivalent to 600 mg curcumin). The use of soluble fibre in the formulation produced a non-digestible gel hydrocolloid that could ferment in the colon, prevent curcumin degradation in the gastrointestinal tract, and retard curcumin release resulting in a lag time of more than 5 h and less than 30% the total release after 24 h [51]. Thus, the fibre–curcumin complex contributed to improved and delayed curcumin absorption [51].

The curcumin formulation named Longvida [44] incorporated solid lipid curcumin particles (SLCP; 650 mg) in their formulation. A single dose of 130–195 mg of curcumin showed a *T*_max_ of 2.4 h. The SLCP is a proprietary formula and comprises of curcumin mixed with soy lecithin containing purified phospholipids, docosahexaenoic acid (DHA), and/or vegetable stearic acid, ascorbyl (vitamin C) esters, and inert ingredients. The improved bioavailability of SLCP compared to unformulated curcumin is linked to key parameters such as curcumin/lipid/antioxidant ratio, globule-size distribution, and stability [51].

Consumption of a combination of curcumin and piperine in the Curcumin C3 Complex + Bioperine resulted in a 20-fold increase in plasma curcumin concentrations compared to the control formulation with the lowest *T*_max_ of 0.69 h. Piperine is a P-glycoprotein and a uridine diphosphate-glucuronosyltransferase (UGT) inhibitor, and is suggested to improve absorption of curcumin by decreasing the efflux in the intestine and increasing the freely available curcumin in the systemic circulation [50]. Although curcumin was not observed in the plasma from 3 to 6 h, the bioavailability improved by 1.5 times compared to that of unformulated curcumin [50].

#### Plasma curcumin concentration in exercise trials

Several studies [22, 23, 25, 52, 53] that investigated the effect of curcumin on exercise-induced inflammatory and oxidative stress markers also evaluated plasma curcumin concentration post-supplementation. All studies [22, 23, 25, 52, 53] observed an increase in plasma curcumin concentration post-supplementation at time points ranging from 2 h to 1–4 days (Table 3). The studies also concluded that supplementation with curcumin resulted in an increase in oxidative capacity [25], improvements in visual analogue scale for muscle soreness [23] and daily analysis of life demands questionnaire [52], and a decrease in CK levels [22, 53] (Table 4).

**Table 3:** Summary of plasma curcumin concentration in studies examining the effect of curcumin intake on exercise-induced muscle damage (EIMD)

Author and year	Population	Duration	Curcumin supplement	Dosage	Plasma curcumin concentration	Sample hydrolysis
Takahashi et al., 2013 [25]	26 males (26.8 ± 2.0 years) (recreationally active)	1 day	Theracurmin	Control	Plasma curcumin concentrations in the double curcumin supplementation trial 2 h after exercise were significantly higher than those in the single curcumin supplementation trial	Hydrolysis
				90 mg/day 2 h before exercise		
				180 mg/day 2 h before and immediately after exercise		
Tanabe et al., 2018 [22]	10 males (28.5 ± 3.4 years) (untrained)	7 days	Theracurmin	Experiment 1—180 mg (90 mg twice a day—at breakfast and dinner) consumed for 7 days before exercise	Plasma curcumin concentrations significantly decreased from baseline (38.8 ± 17.8 ng mL^−1^) to 1, 3, 5, and 7 days after exercise (10.2 ± 5.4, 5.0 ± 2.7, 0.1 ± 0.4, and 0.1 ± 0.2 ng mL^−1^, respectively)	No mention of the process used
	10 males (29.0 ± 3.9 years) (untrained)			Experiment 2—180 mg (90 mg twice a day—at breakfast and dinner) Consumed for 7 days after exercise	Plasma curcumin significantly increased from baseline (5.3 ± 6.1 ng mL^−1^) to 1 day (50.6 ± 25.6 ng mL^−1^), and then maintained a high plasma curcumin concentration through 3, 5, and 7 days after exercise (58.5 ± 37.1, 42.2 ± 46.0, and 41.1 ± 29.2 ng mL^−1^, respectively)	
Tanabe et al., 2019 [23]	8 males (28.0 ± 3.2 years) (untrained)	7 days	Theracurmin CR-033P	Control	–	Hydrolysis
	8 males (28.8 ± 3.6 years) (untrained)			PRE—180 mg (90 mg twice a day—at breakfast and dinner) Consumed for 7 days before exercise	At baseline and at 1–3 d after exercise, the plasma curcumin concentration of the PRE group was significantly higher than that in the control and POST groups	
	8 males (29.8 ± 3.4 years) (untrained)			POST—180 mg (90 mg twice a day—at breakfast and dinner) Consumed for 4 days after exercise	At 1–4 d after exercise, the plasma curcumin concentration of the POST group was significantly higher than that of the control and PRE groups	
Tanabe et al., 2015 [53]	14 males (23.5 ± 2.3 years) (UNTRAINED)	Single dose	Theracurmin	300 mg 150 mg—1 h before exercise 150 mg—12 h after exercise	Plasma curcumin concentration increased from baseline (0.04 ± 0.07 ng/mL) to 127.7 ± 144.6, 85.7 ± 51.6, 8.6 ± 5.5, 2.2 ± 1.4 and 0.9 ± 0.6 ng/mL at 0, 24, 48, 72 and 96 h after exercise, respectively	No mention of the process used
Sciberras et al., 2015 [52]	11 males (35.5 ± 5.7 years) (trained)	4 days	Meriva®	500 mg with midday meal for 3 days and 500 mg just before exercise	Mean ± SD (range) curcumin concentration obtained was 79.7 ± 26.3 ng/ml (50.7 ng/mL to 125.5 ng/mL)	Hydrolysis

**Table 4:** Summary of studies examining the effect of curcumin intake on exercise-induced muscle damage (EIMD)

Author and year	Population	Study design	Duration	Curcumin supplement	Dosage	Curcuminoids content	Activity type	Key findings
Takahashi et al., 2013 [25]	26 males (26.8 ± 2.0 years) (recreationally active)	Double-blind, placebo-controlled, counterbalanced crossover design	1 day	Theracurmin	Placebo	NA	Walking or running at 65% V̇O_2_ max on a treadmill for 60 min	↑ GPX ↑ d-ROM’s
					90 mg/day 2 h before exercise	10% curcumin, 2% curcuminoids without curcumin		↑ Plasma curcumin ↑ BAP ↑ GSH † TBARS † GSSH † SOD
					180 mg/day 2 h before and immediately after exercise			
Nakhostin-Roohi et al., 2016 [19]	10 males (25.0 ± 1.6 years) (untrained)	Randomised controlled trial—double-blind crossover	Single dose	Theracurmin	150 mg Immediately post-exercise	10% curcumin, 2% curcuminoids without curcumin	Unaccustomed squat exercises	↓ CK ↓ VAS for pain ↑ TAC
Tanabe et al., 2018 [22]	10 males (28.5 ± 3.4 years) (untrained)	Double‐blind crossover design	7 days	Theracurmin	180 mg (90 mg twice a day—at breakfast and dinner) Consumed for 7 days before exercise	Not defined	30 maximal eccentric contractions of the elbow flexors at an angular velocity of 120°/s	↓ Plasma curcumin ↓IL-8
	10 males (29.0 ± 3.9 years) (untrained)				180 mg (90 mg twice a day—at breakfast and dinner) Consumed for 7 days after exercise			↑ Plasma curcumin ↓ VAS for muscle soreness ↓ CK
Tanabe et al., 2019 [23]	8 males (28.8 ± 3.6 years) (untrained)	Randomised, controlled, single-blind, parallel design study	7 days	Theracurmin CR-033P	PRE—180 mg (90 mg twice a day—at breakfast and dinner) Consumed for 7 days before exercise	30% curcumin, 6% other curcuminoids	30 maximal eccentric contractions of the elbow flexors at an angular velocity of 120°/s	↑ Plasma curcumin at baseline † CK
	8 males (29.8 ± 3.4 years) (untrained)		4 days		POST—180 mg (90 mg twice a day—at breakfast and dinner) Consumed for 4 days after exercise			↓ VAS for muscle soreness † CK
Tanabe et al., 2015 [53]	14 males (23.5 ± 2.3 years) (untrained)	Randomised, crossover design	Single dose	Theracurmin	300 mg 150 mg—1 h before exercise 150 mg—12 h after exercise	Not defined	50 maximal eccentric contractions of the elbow flexors at an angular velocity 120°/s	↑ Plasma curcumin ↓ MVC torque ↓ CK † IL-6 and TNF-*α*
McFarlin et al., 2016 [24]	16 (5 M, 11 F) (20 ± 1 years) (untrained)	Randomised controlled	6 days	Longvida	400 mg 48 h before exercise and for 72 h after	Not defined	6 sets of 10 repetitions of the leg press exercise with a beginning load set at 110% of their estimated 1RM	† Subjective quadriceps pain †ADL ↓CK ↓TNF-*α* ↓IL-8 † IL-10 † IL-6
Sciberras et al., 2015 [52]	11 males (35.5 ± 5.7 years) (trained)	Double-blind randomised crossover	4 days	Meriva®	500 mg with midday meal for 3 days and 500 mg just before exercise	Not defined	Participants exercised for 2 h at a power output equivalent to 95% of their lactate threshold	† IL-6, IL-10 ↑ DALDA Questionnaire (better than usual)
F. Drobnic et al., 2014 [70]	C—9 males (32.7 ± 12.3 years) (trained)	Randomised, placebo-controlled, single-centre, single-blind pilot trial	4 days	Meriva®	1 g twice a day 48 h prior to exercise and continued for 24 h after exercise	200 mg/dose	Modified downhill running test—running at a constant speed for 45 min after a 10-min warm-up on treadmill	†CRP † hsCRP † MCP-1 † FRAP † GPx † CK ↓ IL-8 † Intensity of pain
	P—10 males (38.1 ± 11.1 years) (trained)							
Jäger et al., 2019 [57]	63 (31 M, 32 F) (21 ± 2 years) (trained)	Double-blind, randomised, placebo-controlled parallel design	8 weeks	CurcuWIN®	Low-dose group 250 mg thrice a day (breakfast/lunch/dinner)	50 mg of curcuminoids	45-min downhill run at a – 15% grade and speed equivalent to 65% VO_2_Max after 5-min warm up	↑ Subjective muscle pain (anterior, posterior) all groups † Subjective (total) muscle pain in high-dose group 1 h and 24 h post-exercise † Maximum bending torque and bending power in low-dose group
					High-dose group 1000 mg thrice a day (breakfast/lunch/dinner)	200 mg of curcuminoids		
					Placebo	NA		
McAllister et al., 2020 [66]	14 males (21–30 years) (trained)	Double-blinded, randomised, crossover design	4 days	CurcuFresh	1500 mg/day 1000 mg at breakfast and 500 mg at dinner for days 1, 2 and 3 and 45 min before testing on day 4	69 mg of curcuminoids	Dual stress challenges task that consisted of 35 min of steady-state exercise at a workload corresponding to 60% VO_2_ peak with mental stress challenges	† GHS ↓SOD ↓ AOPP ↓ H2O2
S. Basham et al., 2019 [26]	20 males (21.7 ± 2.9 years) (trained)	Randomised, double-blinded, placebo-controlled, crossover	28 days	CurcuFresh	1500 mg/day 1000 mg at breakfast and 500 mg at dinner for 28 days	69 mg of curcuminoids	225 repetitions of sit and stand using an aerobic step bench in 15 min	↓ CK ↓ VAS † TAC † MDA † TNF-*α*
Amalraj et al., 2020 [20]	30 (12 M, 18 F) (36 ± 11 years) (trained)	Randomised, placebo-controlled, double-blind	4 days	Cureit	500 mg/day (consumed on day 2, 3 and 4 of study)	Not defined	Downhill running for 45 min	† CK ↓ VAS for muscle pain
Delecroix et al., 2017 [61]	16 males (20.7 ± 1.4 years) (trained)	Randomised, placebo-controlled, balanced crossover design	4 days	MGD nature	2 g curcumin + 20 mg Piperine consumed three times a day every 6 h between 8 am and 10 pm On exercise day—45 min before, immediately post- and 6 h post-exercise	Not defined	25 repetitions over 25 m of one-leg jumps on an 8% downhill slope	† Isometric peak torque † Concentric peak torque † Jump performance † CK † Muscle soreness at any time point
Herrick et al., 2020 [54]	47 (25 M, 22 F) (21.0 ± 2.6 years) (untrained) G1: 18 (C + FEN) G2: 14 (FEN) G3: 15 (P)	Randomised, double-blind, placebo-controlled, parallel design	28 days	CurQfen	500 mg/day curcumin + 300 mg fenugreek dietary fibre (galactomannans) Consumed in the morning before eating	190 mg of curcumin	Maximal graded exercise test on a cycle ergometer	↑ Physical working capacity at the fatigue threshold ↑ delay the onset of neuromuscular fatigue † V̇O_2_peak †Time to exhaustion
Nicol et al., 2015 [21]	17 males (33.8 ± 5.4 years) (trained)	Double-blind randomised-controlled crossover	5 days	Eurofins scientific inc.	5000 mg/day 5 capsules twice daily for 2.5 days prior to exercise, then 5 capsules twice daily for 2.5 days after exercise	Not defined	7 sets of 10 eccentric single-leg press repetitions on a leg press machine	^a^ ↓ VAS for muscle pain ↓ CK ↓IL-6 (at 24 h relative to baseline) ‡TNF-*α*

↑ Statistically significant increase

↓ Statistically significant decrease

^†^ Change without statistical significance

^‡^ No impact

^a^ moderate to large effect

*C* Curcumin Group, *P* Placebo Group, *M* Males, *F* Females, *GPX* glutathione peroxidase, *d-ROM*’s reactive oxygen metabolites, *BAP* biological antioxidant potential, *GSH* and *GSSH* reduced and oxidised glutathione, *TBARS* thiobarbituric acid-reactive substances, *SOD* superoxide dismutase, *CK* creatine kinase, *VAS* visual analogue scale, *TAC* total antioxidant capacity, *IL-8* Interleukin-8, *MVC* Maximal voluntary contraction, *ADL* Activities of daily living soreness, *TNF-α* Tumour Necrosis Factor-*α*, *IL-10* Interleukin-10, *IL-6* Interleukin-6, *DALDA* Questionnaire Daily Analysis of Life Demands Questionnaire, *CRP* C-Reactive Protein, *MCP-1* monocyte chemoattractant protein-1, *FRAP* Ferric Reducing Antioxidant Power, *AOPP* advanced oxidation protein products, H_2_O_2_ hydrogen peroxide, *MDA* malondialdehyde, *FEN* Fenugreek

It is difficult to compare and evaluate the plasma curcumin concentration as studies by Tanabe et al. [22, 53] did not describe the sample preparation process and studies by Takahashi et al. [25], Sciberras et al. [52], and Tanabe et al. [23] hydrolysed their plasma samples and, therefore, the amount of curcumin in the plasma [48], may have been overestimated.

#### Difficulties in comparing the effects of different pharmacokinetic characteristics of curcumin supplements on muscle damage markers

It is challenging to understand how the measured differences in pharmacokinetic characteristics (*C*_max_, *T*_max_, and AUC) of the specific supplements may relate to changes in inflammatory markers and attenuation of muscle damage. One key contributing factor is that although pharmacokinetic and exercise trials have both been carried out using the same formulations (Theracurmin [41] and Meriva [42]) different doses have been used for the exercise [23, 25, 43] versus the pharmacokinetic studies [41, 42]. In addition, some researchers have not measured the plasma curcumin concentrations post-supplementation, or do not clearly state the curcumin concentrations in the supplement, also making it challenging to compare results [24]. Others have hydrolysed the plasma samples before analysis [23, 25, 43], thus overestimating the true concentration of curcumin in the blood and making it difficult to accurately correlate any observed changes in levels of inflammatory markers to plasma curcumin levels. Finally, although some researchers [54] have reported that curcumin supplementation increased working capacity at the fatigue threshold and delayed the onset of neuromuscular fatigue, they did not analyse common inflammatory markers such as IL-6, TNF-*α*, and CK, also hindering comparisons between studies [54].

### Effect of curcumin on exercise-induced muscle damage

Intense training can lead to EIMD and can cause swelling, reduced ROM, and loss of muscle strength in the affected limb [16–18]. EIMD is characterised by muscular ultrastructural disruption that increases the release of inflammatory cytokines from myofibers and consequently increases their circulating levels. Muscle soreness increases from about 24 to 48 h post-exercise and decreases gradually from 72 h post-exercise. The activity of CK, a marker of EIMD, increases from 24 h onwards post-exercise and is sustained over a period of 7 days post-exercise [30].

Curcumin ingestion results in the attenuation of the release of inflammatory and oxidative markers, muscle pain, muscle performance, and CK levels by modulating inflammatory signalling cascades. Table 4 contains information from studies investigating the effect of curcumin on EIMD. Out of the 15 studies discussed below, the majority of participants in the trials were young physically active males 20–40 years of age. In addition, studies investigating the effect of curcumin supplementation on EIMD employed exercise protocols that led to different levels of muscle damage and assessed a variety of parameters, thus making it difficult to directly compare results and provide definitive conclusions as to whether curcumin supplementation is effective.

#### Muscle soreness

EIMD leads to delayed onset muscle soreness, associated with muscle pain, resulting in reductions in muscle strength and function and impairing physical function for several days post-exercise [55]. Damage to skeletal muscle activates phospholipase A_2_, which leads to the removal of arachidonic acid from the cell membrane [56]. Arachidonic acid is converted to prostaglandin G_2_ (PGG_2_) under the influence of cyclooxygenase (COX-1 and COX-2) and then to prostaglandin H_2_ (a common precursor to all prostaglandins) [27]. Prostaglandins are pro-inflammatory and cause redness, swelling (due to increased membrane permeability), and pain at the site of muscle damage [28]. Curcumin downregulates the expression of COX-2 and thus, decreases the release of prostaglandins [29] which in turn reduces muscle soreness [19–23].

Studies [19–23] have shown significantly lower levels of muscle soreness when curcumin was consumed approximately 24 h before or after exercise. Consumption of 150 mg of curcumin (Theracurmin) immediately post-exercise resulted in a lower visual analogue scale (VAS) score for perceived muscle soreness compared to placebo at 48 and 72 h after unaccustomed squat exercises in untrained males [19]. Similarly, consumption of 180 mg of Theracurmin (90 mg twice a day at breakfast and dinner) for 7 days after exercise resulted in a significant reduction in muscle soreness 3–6 days after eccentric exercise in untrained males compared to the placebo group [22].

A study on untrained males reported a significantly lower perceived muscle soreness VAS score for the upper arm and elbow joint at 3 and 4 days after consumption of 180 mg Theracurmin CR-033P (90 mg twice a day at breakfast and dinner) for 4 days after eccentric elbow flexion [23]. Intake of 500 mg/day (Cureit; consumed on days 2, 3, and 4 after exercise) by trained participants resulted in a decreased pain VAS score from 2.90 to 1.17 in the curcumin group compared to a smaller decrease in the placebo group [20]. Moreover, administration of 2500 mg twice daily of curcumin for 2.5 days before exercise, and then 2500 mg twice daily for 2.5 days after exercise, resulted in moderate to large effect size reductions in muscle pain during single-leg squat and gluteal stretch at 24 and 48 h [21].

Not all studies show clear benefits of curcumin supplementation in reducing muscle soreness. For example, one study implementing 56 days of supplementation with 200 mg of curcuminoids (1000 mg curcumin by CurcuWIN) in trained individuals indicated not statistically significant reductions in muscle soreness. Compared to the placebo and low-dose (50 mg) curcumin groups, the treatment group showed non-significant reductions of 26, 20, and 8% lower muscle soreness immediately, 24, and 48 h post-exercise (conducted on 57 ± 3 days), respectively. Further research would be required to determine if the non-significant recovery benefits from curcumin may have been due to the final supplementation dose being administered 24 h prior to the exercise and the lack of supplementation after exercise [57].

A wide range of curcumin doses were administered across these studies with differences in formulation, frequency, and timing of ingestion relative to exercise, along with the training status of the participants and the exercise protocol used that may have influenced the extent of muscle soreness post-completion of exercise. In addition, VAS for soreness is a subjective measure, varying from individual to individual, thus potentially influencing the results. Nonetheless, studies have reported lower levels of soreness when 180–2500 mg/day curcumin was consumed immediately after exercise and/or within at least 24 h before and/or after exercise [21, 23].

#### Muscle performance

NF-κB is the transcriptional control for myokines (cytokines synthesised and released during muscular contractions) that are involved in post-exercise inflammatory responses [58]. Overuse of joints contributes to high mechanical stress and generates bone and cartilage extracellular matrix fragments that are recognised by receptors expressed by innate immune cells. Cell activation mediated by this process stimulates the activation of NF-κB, resulting in secretion of inflammatory cytokines such as IL-1 and TNF-*α*, which contribute to tissue damage [59]. Curcumin blocks the signalling pathway of NF-κB, reducing the inflammatory response, decreasing swelling, while improving joint mobility and stiffness (assessed via maximum voluntary contraction (MVC) force and ROM) [22, 23, 53].

Intake of 90 mg curcumin (Theracurmin) twice a day (breakfast and dinner) for 7 days in untrained males resulted in significant improvements in MVC torque and ROM compared to the placebo group 3–7 days after eccentric exercise [22]. In a similar study, consumption of 90 mg curcumin (TheracurminCR-033P) twice a day (breakfast and dinner) for 4 days after eccentric exercise resulted in improvements in ROM of the elbow joint at 3–4 days in untrained males compared to the placebo group. The increases in the degree of ROM coincided with improvements in muscle soreness, indicating that the two could be related. However, another study [23] involving 30 maximal eccentric contractions of elbow flexors showed no significant differences in MVC torque between curcumin and placebo groups at all time points [23]. Muscle regeneration begins on day 3 after exercise [60], and since the follow-up of the recovery period was only 4 days, this time period may have been insufficient to establish the effect of curcumin on MVC torque [23].

One study examining the effect of 50 mg of curcumin (in the form of 250 mg of CurcuWIN®) or 200 mg of curcumin (in the form of 1000 mg of CurcuWIN®) over 56 days in physically active men and women reported that curcumin could prevent decreases in peak extension torque values observed at 1 and 24 h after muscle-damaging exercise (downhill running) [57]. However, changes in isokinetic peak and average flexion torque, peak extension and flexion power, peak and average peak torque failed to yield statistical significance at 1, 24, 48, and 72 h post-exercise. One of the limitations of the supplementation protocol used was conducting the exercise protocol after discontinuing curcumin supplementation and it is possible that continuing supplementation may have prevented a decrease in the performance measures [57].

Ingestion of 150 mg of curcumin (Theracurmin) immediately and 12 h after eccentric exercise in untrained males led to a decrease in MVC from baseline [53]. However, the decrease in MVC was significantly smaller (33.0 ± 8.0%) in the curcumin group than in the placebo group (40.0 ± 9.1%) immediately after exercise and 48–96 h after exercise. The smaller decrease in MVC indicates that intake of curcumin leads to a lower level of muscle damage than the placebo group. However, ROM significantly decreased through all time points from the baseline in the curcumin group and there was no significant interaction effect for the changes compared to the placebo groups [53]. This suggests that supplementation with 150 mg Theracurmin twice a day for only one day is inadequate to improve ROM post-exercise.

In a randomised crossover trial, elite rugby players, consuming 6 g of curcumin and 60 mg of piperine (MGD Nature, Brandérion, France) each day starting 48 h pre-exercise and continuing until 48 h post-exercise experienced a moderately smaller loss of mean power output during the 6-s sprint compared to the control group 24 h after exercise [61]. However, this result was counterbalanced by the absence of effect from curcumin supplementation on isometric peak torque, concentric peak torque, and jump performance at all time points. This was possibly because the EIMD protocol consisted of 25 repetitions over 25 m of one-leg jumps on an 8% downhill slope, which required a greater neuromuscular recruitment pattern compared to isokinetic and isometric tests [61]. Furthermore, a possible limitation of this study could be the quick muscle damage recovery kinetics (training adaptations) which could be a result of the population tested, i.e. elite rugby players [61].

Another study examining the effects of curcumin in combination with fenugreek soluble fibre (CUR + FEN) or fenugreek soluble fibre alone (FEN) on the physical working capacity at the fatigue threshold (PW_CFT_), peak oxygen consumption (V̇O_2_ peak), and time to exhaustion (*T*_lim_) on a graded exercise test in untrained subjects for 28 days [54], showed no effect of curcumin supplementation on V̇O_2_ peak or *T*_lim_. However, the PW_CFT_ was greater after combined supplementation with curcumin and FEN compared with a placebo in ~20% of subjects [54].

Thus, based on the results obtained by Tanabe et al. [23], improvements in both MVC force and ROM in untrained males could be a result of consumption of 180 mg of curcumin in divided doses (two times a day) across 4–7 days post-eccentric exercise [23].

#### Creatine kinase

Muscle creatine kinase (CK-MM), a marker of EIMD, is one of the three isoforms of CK, and is present at places within the muscle fibre where ATP consumption is high [62]. Eccentric muscle contractions exceeding the muscle’s resistance result in perforations in the sarcolemma and damage to the sarcomeres, leading to increased membrane permeability [30, 62, 63], and release of CK into the interstitial fluid that then enters circulation via the lymphatic system [32]. In addition, the production of prostaglandins under the influence of COX-2 leads to vascular hyperpermeability, which could further aid release of CK [31].

Curcumin supplementation indirectly lowers plasma CK activity in several ways. First, curcumin in the blood offers a membrane-protective effect by altering the structure of the membrane [34] and improving membrane integrity, thus reducing CK release into the blood [64]. Second, curcumin can suppress the regulation of the COX-2 pathway, reducing prostaglandin release and hence influence vascular permeability, ultimately decreasing the intracellular–intravascular flow of CK [31]. Lastly, curcumin’s antioxidant properties can suppress the activity of ROS generated during muscle contractions that would ordinarily contribute to muscle damage via CK release [25, 65].

Several studies [19, 21, 22, 24, 26, 53] have observed significantly lower CK activity in the curcumin supplemented group at doses of 150–5000 mg/day in both trained [21] and untrained individuals [24, 53]. Single-dose investigations with an intake of 150 mg curcumin (Theracurmin) post-eccentric exercise showed a lower rise in CK activity immediately 0, 24, 48, and 72 h post-exercise compared to the placebo group [19]. In addition, CK activity was significantly lower in the curcumin group compared to the placebo group at 24 h [19]. Intake of 180 mg of curcumin (Theracurmin) for 7 days (90 mg twice a day, at breakfast and dinner) after exercise in untrained men also resulted in lower CK activity compared to the placebo group [22]. Consumption of 300 mg curcumin (Theracurmin) in divided doses (150 mg 1 h before exercise and 150 mg 12 h after exercise) showed a significantly smaller peak CK activity for the curcumin group (3398 ± 3562 IU/L) compared to placebo (7684 ± 8959 IU/L) and lower activity at 48, 72 and 96 h compared to baseline CK levels [53]. Similarly, 1500 mg/day (CurcuFresh) of curcumin resulted in significantly lower CK activity (199.62 U/L in the curcumin group compared to 287.03 U/L in the placebo group) [66]. Supplementation with 5000 mg/day of curcumin (Eurofins Scientific Inc.) for 5 days (5 capsules twice daily for 2.5 days before exercise, then 5 capsules twice daily for 2.5 days after exercise) in trained men showed a small reduction in CK activity at 24 and 48 h (−22 to 29%; ±21 to –22%) compared to the baseline values [21]. Lower CK activity in the curcumin groups across the studies may suggest that myofibril damage due to exercise was attenuated by curcumin ingestion [53].

Curcumin supplementation before and/or after exercise can decrease CK activity post-exercise. However, the magnitude of the reduction varies from study to study. Several factors may have contributed to the differing outcomes, including the training status of the participants, exercise protocol, duration of the study, the timing of ingestion of the curcumin supplement, and the formulation of the curcumin supplement itself (including treatments and other ingredients that affect the rate of absorption).

#### Inflammatory markers

Curcumin exerts anti-inflammatory actions by hindering the activation of NF-κB, suppressing the activation and phosphorylation of JAK/STAT proteins, and inhibiting MAPK signalling that contribute to the production of inflammatory markers such as TNF-*α*, IL-6, and IL-8 at the site of muscle damage [35].

*TNF-α*: The effect of curcumin supplementation on reducing TNF-*α* levels in the blood has been evaluated in several studies [21, 22, 24, 26, 53], with equivocal observations reported. No significant differences in plasma TNF-*α* levels were found between the curcumin and placebo trials when 150 mg [53] and 180 mg of curcumin [22] were consumed by untrained males following elbow flexor eccentric exercise [22, 53]. This could be because the exercise protocol involved small muscle mass and was short in duration, and, therefore, did not affect the inflammatory cytokine levels in the blood [67]. In addition, as TNF-*α* has a very short half-life (15–30 min), plasma concentrations do not always reflect those produced by myocytes [68]. Moreover, the TNF-*α* levels were measured in the blood and not the muscle tissue. As concentrations of inflammatory markers and oxidative stress markers after exercise are different between muscle tissue and blood [69], the observation on the effect of curcumin on TNF-*α* levels post-exercise may be limited. In the study by McFarlin et al. [24], untrained subjects completed 6 sets of 10 repetitions of leg press exercise and consumed 400 mg/day of curcumin 48 h before exercise, up until 72 h after exercise. They observed that TNF-*α* levels were significantly lower with curcumin at 1 day (− 25%), 2 days (− 23%), and 4 days (− 23%) compared to placebo and concluded that a minimum dose of 400 mg curcumin could be effective in decreasing circulating levels of TNF-*α* [24].

However, studies involving 1500 mg/day of curcumin supplementation (69 mg of curcuminoids) for 28 days (CurcuFresh) [26] and 5000 mg/day for 5 days (Eurofins Scientific Inc.) [21] reported no significant decrease in plasma TNF-*α* levels after exercise in trained males. Curcumin supplementation in both the studies [21, 26] was likely ineffective because physically active individuals were recruited and the participants’ aerobic training status (150 min of moderate-intensity aerobic activity or 30 min of vigorous-intensity aerobic activity per week) offered the stimulus for adaptations that contributed to lower resting levels of TNF-*α* (1.2 pg/mL), thus negating any potential anti-inflammatory benefits of curcumin supplementation [26]. In addition, the authors proposed that no significant decrease in TNF-*α* levels was observed in the trained individuals because the exercise protocol they used (7 sets of 10 eccentric single-leg press repetitions) failed to cause sufficient muscle damage due to muscle adaptations from prior physical activity [21].

In most studies [21, 22, 53], the exercise protocol used led to only minor increases in plasma TNF-*α* concentrations and reported no significant differences in plasma TNF-*α* levels in curcumin groups compared to placebo groups. Nonetheless, as observed for untrained individuals, a minimum of 400 mg curcumin supplementation before and after eccentric exercise may result in lower increases in TNF-*α* levels post-eccentric exercise [24].

*IL-6 and IL-8* The effect of curcumin supplementation on IL-6 and IL-8 levels before and after exercise in trained and untrained individuals has been evaluated in several studies [21, 22, 24, 53, 70]. No significant decrease in post-exercise IL-6 levels was observed in the curcumin group when compared to placebo groups when supplemented with 300 mg (Theracurmin) [53] or 400 mg of curcumin (Longvida), respectively [24]. However, the exercise protocol chosen (6 sets of 10 repetitions of the leg press exercise with a beginning load set at 110% of their estimated 1-repetition maximum) may not have been sufficient to increase the pro-inflammatory cytokines in the body due to the muscle mass involved and the short duration of the activity [24]. In addition, blood samples were not taken immediately after exercise but were taken at 24, 48, 72, and 96 h post-exercise; therefore, the researchers may have missed observing changes in IL-6 levels which typically increase from 8 to 12 h post-eccentric exercise, and return to baseline levels by 24 h post-exercise [53, 71]. In contrast, Nicol et al. [21] observed a decrease in IL-6 values in the curcumin supplemented group (5000 mg curcumin; Eurofins Scientific Inc.) in trained participants after 7 sets of 10 eccentric single-leg press repetitions on a leg press machine at 24 h relative to immediately post-exercise [21]. However, they also observed an increase in IL-6 levels (small standardised differences) immediately post-exercise (31%; ±29%) and again at 48-h post-exercise (32%; ±29%) relative to baseline, thus, making the overall effect of curcumin supplementation unclear [21].

Supplementation with 180 mg of curcumin for 7 days prior to exercise (30 maximal eccentric contractions of the elbow flexors) in untrained males was associated with significantly lower plasma IL-8 levels 12 h after exercise than the placebo group [22]. This decrease in plasma IL-8 levels was associated with high concentrations of curcumin in the blood during and after exercise that suppressed the exercise-induced inflammatory effect [22]. However, no significant differences were observed when curcumin was ingested after exercise [22]. Conversely, intake of 400 mg/day of curcumin (Longvida) (2 days before exercise and for 3 days after exercise) resulted in a significantly lower IL-8 concentrations at day 1 (−21%) and day 2 (−18%) post-exercise (6 sets of 10 repetitions of leg press) compared to placebo in untrained individuals [24]. Furthermore, intake of 1 g of curcumin supplement (Meriva) twice a day 2 days before exercise and for 1 day after exercise (modified downhill running) in trained individuals also resulted in a significantly lower increase in plasma IL-8 levels 2 h post-exercise [70]. Thus, although the effect of curcumin supplementation on IL-6 levels remains unclear, curcumin intake before and after exercise may lower serum IL-8 levels in both trained and untrained individuals.

#### Oxidative markers

Reactive oxygen species are produced by a variety of extracellular and intracellular agents such as electron leakage from the mitochondrial respiratory chain and NADPH oxidases [72]. Exercise can induce oxidative stress by increasing oxygen utilisation up to 200-fold in active muscles and contributing to excessive amounts of ROS [73, 74], which can damage DNA, proteins, and lipids [75, 76] and affect exercise performance [77].

Reactive oxygen species contribute to oxidative stress and maintain inflammation by promoting the activation of NF-κB. During a sustained inflammatory response, accumulation of neutrophils within tissues provides a growth medium for producing oxidative enzymes, cytokines, and chemokines [78–80]. Curcumin can suppress the activation of NF-κB and potentially lead to elevated antioxidant responses by activating NRF2 [36], which upregulates the synthesis of antioxidant proteins that protect against oxidative damage triggered by injury and inflammation [37]. Thus, activation of NRF2 can improve the total antioxidant capacity of the body and reduce the harmful effects of ROS [36]. In addition, the phenolic OH group of curcumin has the potential to act as a ROS scavenger and a quencher of the lipid peroxidative side chain, thus reducing the activity of lipid hydroperoxides [81].

Curcumin supplementation (Theracurmin) with 90 mg/day (2 h before endurance exercise) and 180 mg/day (90 mg 2 h before and immediately after endurance exercise) in healthy men attenuated exercise-induced increases in the serum concentrations of derivatives of reactive oxygen metabolites (d-ROMs) and serum biological antioxidant potential (BAP), and also reduced plasma glutathione levels (GSH) post-exercise [25]. However, there were no significant increase observed in superoxide dismutase (SOD), and glutathione peroxidase (GPx) concentrations immediately after and post-2 h of exercise compared to the pre-values in both single and double curcumin supplementation groups [25]. In addition, supplementation with 150 mg curcumin (Theracurmin) after squat exercises in untrained males resulted in improved total antioxidant capacity (TAC) at 24 and 48 h post-exercise compared to the placebo group [19].

In a study [22] where curcumin (180 mg; Theracurmin) was supplemented for 7 days before and after exercise in a double-blind crossover study, no statistically significant improvements in serum concentrations of d-ROMs and BAP were observed in the curcumin and placebo groups as well as between groups over time [17]. It is possible that the exercise protocol used in this study (30 maximal eccentric contractions of the elbow flexors at an angular velocity of 120°/s) was insufficient to result in significant oxidative stress [22]. Similarly, curcumin supplementation (1500 mg/day (1000 mg breakfast, 500 mg dinner); CurcuFresh) for 28 days in trained males showed no significant improvements in TAC post-exercise (225 repetitions of sit and stand using aerobic step bench over 15 min) compared to the placebo. According to the authors, the lack of significant changes in TAC may be because the assay did not quantify changes in enzymatic antioxidants and had poor sensitivity [26].

In a trial comparing the effects of curcumin (1 g, Meriva, 2× per day, 2 days before, and 1 day after exercise) versus placebo on catalase (CAT) and GPx levels in aerobically trained males who completed a 2 h downhill run, levels of both enzymes tended to increase 2 h after exercise and returned towards baseline values 24 h after exercise in both curcumin and placebo groups [70]. The recruitment of aerobically trained participants may have limited muscle damage with the exercise protocol used, thus possibly explaining limited changes in CAT and GPx activity [65].

Thus, results from two studies indicate that supplementation with 90–180 mg curcumin 2 h before exercise or immediately after exercise may improve the antioxidant capacity of the body [19, 25]. However, more quality research is needed to clarify the optimal dose.

Based on the studies discussed in this review, curcumin is beneficial in alleviating exercised-induced muscle damage. However, due to the vast differences in the formulations of curcumin supplements and study protocols, it is difficult to determine a single dose that would be effective in reducing various inflammatory markers and increase the antioxidant enzymes such as SOD and GPx. Table 5 summarises the timing and dose of curcumin supplementation required to improve muscle soreness and performance, inflammatory markers, and oxidative markers associated with EIMD in trained and untrained participants after eccentric and endurance exercise.

**Table 5:** Summary of the timing and dose of curcumin supplementation required to improve muscle soreness and performance, inflammatory markers, and oxidative markers associated with exercise-induced muscle damage (EIMD)

EIMD markers and oxidative markers	Author	Training status of participants	Exercise protocol	Formulation	Curcuminoids content	Duration	Dosage	Timing of dose
Muscle soreness	Nakhostin-Roohi et al., 2016 [19]	Untrained	Unaccustomed squat exercises	Theracurmin	10% curcumin, 2% curcuminoids without curcumin	Single dose	150 mg	Immediately post-exercise
	Tanabe et al., 2018 [22]	Untrained	30 maximal eccentric contractions of the elbow flexors at an angular velocity of 120°/s	Theracurmin	Not defined	7 days	180 mg	90 mg twice a day at breakfast and dinner, consumed for 7 days after exercise
	Tanabe et al., 2019 [23]	Untrained	30 maximal eccentric contractions of the elbow flexors at an angular velocity of 120°/s	Theracurmin	30% curcumin, 6% other curcuminoids	4 days	180 mg	90 mg twice a day at breakfast and dinner, consumed for 4 days after exercise
	Amalraj et al., 2020 [20]	Trained	Downhill running for 45 min	Cureit	Not defined	4 days	500 mg/day	Consumed on day 2, 3 and 4 of study
	Nicol et al., 2015 [21]	Trained	7 sets of 10 eccentric single-leg press repetitions on a leg press machine	Eurofins scientific Inc	Not defined	5 days	5000 mg/day	5 capsules (2.5 g curcumin) twice daily for 2.5 days prior to exercise, then 5 capsules twice daily for 2.5 days after exercise
Muscle performance	Tanabe et al., 2018 [22]	Untrained	30 maximal eccentric contractions of the elbow flexors at an angular velocity of 120°/s	Theracurmin	Not defined	7 days	180 mg	90 mg twice a day at breakfast and dinner, consumed for 7 days after exercise
	Tanabe et al., 2019 [23]	Untrained	30 maximal eccentric contractions of the elbow flexors at an angular velocity of 120°/s	Theracurmin	30% curcumin, 6% other curcuminoids	4 days	180 mg	90 mg twice a day at breakfast and dinner, consumed for 4 days after exercise
	Tanabe et al., 2015 [53]	Untrained	50 maximal eccentric contractions of the elbow flexors at an angular velocity 120°/s	Theracurmin	Not defined	Single dose	300 mg	150 mg 1 h before exercise and 150 mg 12 h after exercise
Creatine kinase	Nakhostin-Roohi et al., 2016 [19]	Untrained	Unaccustomed squat exercises	Theracurmin	10% curcumin, 2% curcuminoids without curcumin	Single dose	150 mg	Immediately post-exercise
	Tanabe et al., 2018 [22]	Untrained	30 maximal eccentric contractions of the elbow flexors at an angular velocity of 120°/s	Theracurmin	Not defined	7 days	180 mg	90 mg twice a day at breakfast and dinner, consumed for 7 days after exercise
	McFarlin et al., 2016 [24]	Untrained	6 sets of 10 repetitions of the leg press exercise with a beginning load set at 110% of their estimated 1RM	Longvida	Not defined	6 days	400 mg	48 h before exercise and for 72 h after
	Tanabe et al., 2015 [53]	Untrained	50 maximal eccentric contractions of the elbow flexors at an angular velocity 120°/s	Theracurmin	Not defined	Single dose	300 mg	150 mg 1 h before exercise and 150 mg 12 h after exercise
	S. Basham et al., 2019 [26]	Trained	225 repetitions of sit and stand using an aerobic step bench in 15 min	CurcuFresh	69 mg of curcuminoids	28 days	1500 mg/day	1000 mg at breakfast and 500 mg at dinner
	Nicol et al., 2015 [21]	Trained	7 sets of 10 eccentric single-leg press repetitions on a leg press machine	Eurofins scientific Inc	Not defined	5 days	5000 mg/day	5 capsules (2.5 g curcumin) twice daily for 2.5 days prior to exercise, then 5 capsules twice daily for 2.5 days after exercise
Tumour necrosis factor-*α*	McFarlin et al., 2016 [24]	Untrained	6 sets of 10 repetitions of the leg press exercise with a beginning load set at 110% of their estimated 1RM	Longvida	Not defined	6 days	400 mg	48 h before exercise and for 72 h after
Interleukin-6	Nicol et al., 2015 [21]	Trained	7 sets of 10 eccentric single-leg press repetitions on a leg press machine	Eurofins scientific Inc	Not defined	5 days	5000 mg/day	5 capsules (2.5 g curcumin) twice daily for 2.5 days prior to exercise, then 5 capsules twice daily for 2.5 days after exercise
Interleukin-8	McFarlin et al., 2016 [24]	Untrained	6 sets of 10 repetitions of the leg press exercise with a beginning load set at 110% of their estimated 1RM	Longvida	Not defined	6 days	400 mg	48 h before exercise and for 72 h after
	F. Drobnic et al., 2014 [70]	Trained	Modified downhill running test—running at a constant speed for 45 min after a 10-min warm-up on treadmill	Meriva®	200 mg/dose	4 days	1 g twice a day	48 h prior to exercise and continued for 24 h after exercise
Biological antioxidant potential and glutathione	Takahashi et al., 2013 [25]	Recreationally active	Walking or running at 65% V̇O_2_ max on a treadmill for 60 min	Theracurmin	10% curcumin, 2% curcuminoids without curcumin	1 day	180 mg/day	2 h before and immediately after exercise

### Limitations of research and future directions

The curcumin formulations discussed in this review have been developed with varying amounts of curcuminoids and different ingredients that impact their bioavailability, thus making it complicated to suggest a single optimal dose for reducing inflammation post-exercise. However, formulation-specific doses can be suggested based on the scientific research for each particular product.

Most of the studies discussed in this review recruited young healthy participants and there are no data available for the effect of curcumin on EIMD in older adults. Older adults are of special interest as they experience sarcopenia: generalised loss of skeletal muscle mass and muscle strength with age. As a result, older individuals have low muscle mass, low muscle strength, and an increased body fat percentage that contributes to chronic inflammation and oxidative stress compared to young individuals [82]. Moreover, the body composition of older individuals with sarcopenia is significantly altered compared to that of a young population. Therefore, the results from the studies based on the effect of curcumin on EIMD in young trained and untrained individuals should not be extrapolated to a sarcopenic older population. In addition, research on the chronic effects of curcumin consumption based on specific formulations, in association with an appropriate exercise protocol are required to evaluate their effects on recovery from EIMD.

## Conclusion

Curcumin is a challenging ergogenic aid to study due to its poor bioavailability and poor metabolism in the intestine and liver. However, new formulations using nanoparticles [41], phytosomes [42], micelles [43], and phospholipid complexes [44] have been developed which demonstrate improved bioavailability and their effects on EIMD have been investigated in humans. Overall, curcumin supplementation is most effective in reducing EIMD when consumed by untrained individuals. Supplementation immediately after exercise and/or within at least 24 h before and/or after exercise is highly recommended. Though the optimum amount of curcumin required to decrease serum IL-6 levels is still unclear, supplementation with 400–1000 mg of curcumin 1–2 times a day could be considered to aid in improving muscle performance and lowering circulating IL-8 levels. To date, one study in untrained males has shown decreases in TNF-*α* levels post-exercise following consumption of 400 mg/day of curcumin for a period of 6 days [24]; however, more studies are needed to confirm this effect. In addition, curcumin supplementation in the range of 150–5000 mg/day has been effective in decreasing CK levels in both untrained and trained individuals when consumed pre- and/or post-exercise. To improve the antioxidant capacity of the body post-exercise, curcumin supplementation with 90–180 mg curcumin 2 h before exercise or immediately after exercise may be effective [19, 25].

Curcumin impacts the production of several exercise-induced inflammatory markers. However, the amount of curcumin required to elicit changes in these inflammatory markers vary significantly. In addition, as no two studies follow the same protocol along with the same curcumin formulation, it is challenging to conclude the amount of curcumin required to reduce EIMD.

## Data Availability

Not applicable.
